# Hemoglobin-based oxygen carriers, oxidative stress and myocardial infarction

**DOI:** 10.3389/fphys.2025.1551932

**Published:** 2025-04-15

**Authors:** Timothy N. Estep

**Affiliations:** Chart Biotech Consulting, LLC, Erie, CO, United States

**Keywords:** hemoglobin-based oxygen carriers, HBOC, myocardial infarction, oxidative stress, nitric oxide, hemoglobin autoxidation, pharmacokinetics, AUC

## Abstract

**Introduction:**

Development of hemoglobin-based oxygen carriers (HBOCs) for use as temporary blood replacement solutions and treatment of hemorrhagic shock has been hindered because of evidence HBOC infusion increases the risk of myocardial infarction (MI).

**Methods:**

To gain insight into potential toxicity mechanisms, MI incidence from later stage clinical testing of five HBOCs was compared to pharmacokinetic and biochemical parameters to identify correlations suggestive of cause-and-effect hypotheses.

**Results:**

There are positive correlations between MI incidence and HBOC dose, size, intravascular half-life and area under the plasma concentration versus time curve (AUC). Furthermore, MI incidence is positively correlated with initial rates of HBOC autoxidation, oxidation by nitric oxide, and AUCs estimated for these HBOC oxidation products.

**Conclusions:**

These observations imply that increased MI risk after HBOC infusion is due to intravascular reactions which exacerbate oxidative stress.

## Introduction

Hemoglobin-based oxygen carriers (HBOCs) have been under development for several decades to improve trauma resuscitation outcomes and provide alternative oxygen transport solutions when blood is not available (Liu et al., 2022). While the efficacy of such solutions has been demonstrated in preclinical studies and human patients, regulatory approval has yet to be obtained in most countries due to concerns about serious adverse events (Estep, 2019). Such concerns were crystallized by a meta-analysis demonstrating increased risk of mortality and myocardial infarction (MI) after HBOC infusion (Natanson et al., 2008).

To develop hypotheses as to potential mechanism(s) of MI risk enhancement, the preclinical literature on HBOC toxicity and clinical data collected during later stage testing were reviewed. Both *in vitro* and *in vivo* experiments suggest that HBOCs may increase toxicity by exacerbating oxidative stress in a synergistic fashion with other insults (Alayash, 2019), particularly with respect to the endothelium (Biro, 2012). There is also an extensive clinical literature implicating oxidative stress as a risk factor for MI (Wang and Kang, 2020).

No single clinical trial performed with HBOCs was adequately powered to assess differences in serious adverse events on the order of a few percent. Thus, aggregation of data is required to identify significant correlations (Natanson et al., 2008). In the present analysis, the ratio of MI incidence in treated *versus* control patients was calculated from published clinical data for four crosslinked and/or polymerized HBOCs (CP HBOCs), HemAssist^™^, Hemolink^™^, Hemopure® and Polyheme^™^, and one polyethylene derivatized hemoglobin HBOC (PEG HBOC), Hemospan® (also denoted as MP-4) (Przybelski et al., 1999; Sloan et al., 1999; Garrioch et al., 1999; Lamy et al., 2000; Schubert et al., 2002; Kerner et al., 2003; Bloomfield et al., 2004; Schubert et al., 2003; Cheng, 2001; Hill et al., 2002; Cheng et al., 2002; Greenburg and Kim, 2004; Jahr et al., 2008; Kasper et al., 1996; Kasper et al., 1998; Standl et al., 1998; LaMuraglia et al., 2000; Levy et al., 2002; Sprung et al., 2002; Hemelrijck et al., 2014; Gould et al., 1998; Northfield Laboratories, 2017; Moore et al., 2009; Olofsson et al., 2006; Olofsson et al., 2008; Olofsson et al., 2011; Van der Linden et al., 2011), and compared to a variety of parameters (Meng et al., 2018) to characterize the pharmacokinetics and toxicodynamics with respect to MI. All of the evaluated HBOCs use mammalian tetrameric hemoglobin as the oxygen transporting component, four human and one (Hemopure) bovine (Table 1). Due to their similarities with respect to the structure of the hemoglobin starting material, size of chemical modification reagents, rates of reaction with nitric oxide (NO), and oxygen binding characteristics, the CP HBOCs were analyzed as a subgroup. Hemospan is neither crosslinked nor polymerized. In addition, to better define correlations with specific reaction products, a mathematical model of the evolution of total, reduced, autoxidized and nitic oxide oxidized HBOC species was developed to identify the most important reactions in the etiology of MI risk enhancement.

**TABLE 1:** Properties of HBOCs analyzed.

HBOC	HemAssist	Hemolink	Hemopure	Hemospan	PolyHeme
Hb Species	Human	Human	Bovine	Human	Human
Modification	Crosslinked	Crosslinked/Polymerized	Crosslinked/Polymerized	PEG Derivatized	Crosslinked/Polymerized
MW Range (kDa)^a^	64–128	64–500	130–500	95	130–250
P_50_ (mmHg)^a^	31.1	34.4	34.3	8.2	31.3
Avg Radius of Gyration (nm)^b^	3.1	4.9	7.5 (est.)	9.3	8.9 (est.)
MI Incidence^c^					
Treated	6/402	20/206	6/592	8/464	21/451
Fractional incidence	0.0149	0.0971	0.0201	0.0172	0.0466
99% CI	0.0027 0.393	0.0518 0.1606	0.0018 0.0268	0.0049 0.0401	0.0249 0.0781
Controls	5/398	15/213	2/531	1/428	3/459
Fractional incidence	0.0126	0.0704	0.0038	0.0023	0.0065
99% CI	0.0014 0.0360	0.0328 0.1272	−0.0017 0.0187	−0.0029 0.0193	−0.0010 0.0250
MI Incidence Ratio^d^	1.19	1.38	2.69	7.38	7.12

^a^ Data from (Meng et al., 2018).

^b^ Data from (Vandegriff et al., 1997; Vandegriff et al., 2003) or estimated as described in Methods.

^c^ Number of patients with MI/total number of patients.

^d^ Quotient of MI, incidence in treated patients divided by MI, incidence in controls.

## Methods

### Calculation of MI incidence ratio

After an extensive literature search, MI data were tabulated from all of the randomized, controlled Phase II and III clinical trials (RCTs) of HBOCs used in the treatment of surgical blood loss or trauma resuscitation wherein the total patients in each of the treated and control groups exceeded 200. The five HBOCs meeting these criteria are noted in the Introduction. While adjudicated MI data were reported in one clinical trial (Moore et al., 2009), only the MI incidences as assessed by the physicians directly treating patients were used in this analysis for consistency of comparison. The MI ratio for each HBOC was calculated by dividing the MI incidence rate in treated patients by the incidence rate in controls (Table 1). This procedure was used to adjust for the facts that differing numbers of patients were enrolled in treated and control groups and the variation of MI incidence between different control patient populations was significant.

### Calculation of average HBOC dose and size

Average HBOC doses ([Hb]_0_, g/kg) were calculated as number weighted averages of the average doses utilized in RCTs with a particular HBOC. When doses were not reported as g/kg, they were estimated by division of the average total dose by average patient weight. If average patient weights were not reported, they were estimated using continent/country specific averages (North America 80.7 kg, Europe 70.8 kg South Africa 73.0 kg) (Walpole et al., 2012; WorldData.info., 2023). The average dose of Polyheme in a trial using repetitive stepwise hemodilution was corrected for the estimated product loss due to blood removal after the first HBOC dose was administered. Average doses in g/kg were converted to heme concentration (mM/L) by multiplying by the conversion factor 1.47, which assumes a plasma volume of 42 mL/kg. Average molecular size (radius of gyration, R_g_) was based on published values (Vandegriff et al., 1997; Vandegriff et al., 2003) or estimated by assuming Rg is equivalent to a linear right circular cylinder composed of the number of hemoglobin tetramers equivalent to the average molecular weight listed in Table 1. This method was selected amongst several evaluated because it accurately reproduced the measured Rg for Hemolink (5.0 nm calculated *versus* 4.9 measured).

### Calculation of estimated AUC

**FIGURE 1:**
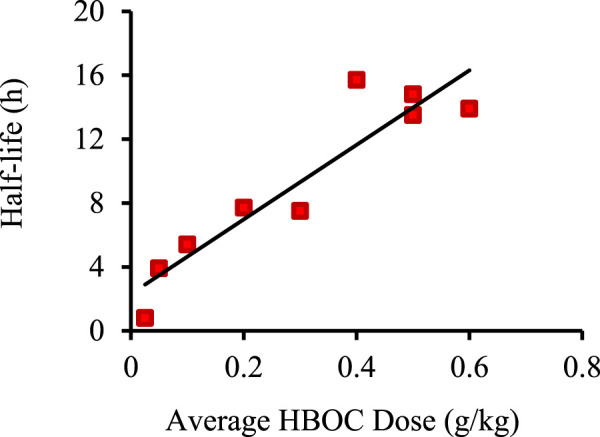
Example of best linear fit of HBOC plasma half-life *versus* dose using data for Hemolink. Data taken from (Carmichael et al., 2000). Linear best fit was determined using the Excel data analysis package.

Assuming that HBOC plasma clearance is adequately described as a single exponential decay (Olofsson et al., 2006; Olofsson et al., 2008; Estep, 2019; Carmichael et al., 2000; Swan et al., 1995; O’Hare et al., 2001; Hughes et al., 1996), integration leads to the expression:

$$\text{AUC}=1.443{\left[\text{Hb}\right]}_{0}T_{1/2}$$ (1)

where T_1/2_ is the circulatory half-life. The functional dependence of T_1/2_ on [Hb]_0_ was estimated for each HBOC (Table 2; Figure 1) as the best linear fit to published T_1/2_ *versus* dose data (Standl et al., 1998; Olofsson et al., 2006; Olofsson et al., 2008; Carmichael et al., 2000; Przybelski et al., 1996; Swan et al., 1995; O’Hare et al., 2001; Hughes et al., 1995; Hughes et al., 1996). The resulting equation was substituted into (Equation 1) to yield an estimated AUC as a quadratic function of [Hb]_0_ of the form:

$$\text{AUC}=1.443\left[{\text{Hb}}_{0}\right]\left(A{\left[\text{Hb}\right]}_{0}+B\right)$$

where A and B are coefficients derived from the best linear fit to the T_1/2_ *versus* dose plots. Due to the lack of T_1/2_ *versus* dose data for Polyheme, the corresponding dependence for Hemopure was utilized, since Hemopure is the most similar HBOC of those evaluated to Polyheme with respect to overall structure.

**TABLE 2:** Properties of equations of best linear fit of half-life *versus* dose data for HBOCs.^a^.

HBOC	Equation of best fit T_1/2_ (h), [Hb]_0_ (g/kg)	R^2^	p value	n	T_1/2_ at Avg Dose (h)
HemAssist	T_1/2_ = 8.4 [Hb]_0_ + 2.50	0.9632	0.006	7	8.2
Hemolink	T_1/2_ = 23.3 [Hb]_0_ + 2.31	0.8619	0.001	9	23.1
Hemopure	T_1/2_ = 22.8 [Hb]_0_ + 3.54	0.7287	0.029	7	37.0
Hemospan	T_1/2_ = 16.2 [Hb]_0_ + 14.3	0.5333	0.125	7	18
Polyheme^b^					53.2

^a^ Linear best fits determined using Excel data analysis package. P values are two tailed.

^b^ Due to the lack of dose *versus* half-life data for Polyheme, the best fit equation for Hemopure was utilized for this calculation.

### Sources and calculations of other HBOC biophysical and biochemical parameters

HBOC biophysical and biochemical parameters were taken from the compendium of Meng and coworkers (Meng et al., 2018; Table 3). Initial HBOC autoxidation reaction rates were estimated by multiplying the autoxidation rate constant (k_a_) by [Hb]_0_. The initial rate of hemoglobin reaction with nitric oxide (NO) was calculated by multiplying [Hb]_0_ with the corresponding reaction rate constant (k_N_) assuming an initial NO concentration of 1 nM, a value within the range of plasma NO concentrations derived from multiple studies (Hall and Garthwaite, 2009). Quasi steady-state NO concentrations were calculated using an average NO synthesis rate of 1.7 mmoles/day (Hall and Garthwaite, 2009). Assuming that half is secreted into an average of 3 L of plasma, this equates to approximately 0.01 mM/L/h of NO secretion. Due to the rapidity of the hemoglobin reaction with NO, it was assumed that any secreted NO reacts immediately with plasma HBOC. These assumptions lead to the result that the steady state concentration of NO after the administration of each HBOC, ([NO]_ss_) is given by:

$${\left[\text{NO}\right]}_{\text{ss}}=0.01/{\left[\text{Hb}\right]}_{0}/k_{N}$$

**TABLE 3:** Average HBOC doses, reaction rate constants and initial reaction rates.

HBOC	HemAssist	Hemolink	Hemopure	Hemospan	PolyHeme
[Hb]_0_ (g/kg)^a^	0.68	0.89	1.47	0.23	2.18
[Heme]_0_ (mM heme/L)^b^	1.00	1.31	2.17	0.34	3.21
Calculated half-life for dose [Hb]_0_ (h)^c^	8.2	23.1	37.0	18.0	53.2
AUC (mM heme x h/L)^d^	11.8	43.6	116.2	8.8	246.4
k_a_ (h^-1^)^e^	0.081	0.130	0.220	0.070	0.260
k_T_ (h^-1^)^f^	0.084	0.030	0.019	0.039	0.013
k_N_ (mM^-1^h^-1^)^g^ x10^−8^	1.49	1.34	1.58	1.60	1.49
k_hemelossfast_ ^e^	7.30	7.30	3.47	14.80	11.70
[NO]_ss_ (mM/L)^h^ x10^10^	1.50	1.28	0.66	4.21	0.47
Initial reaction rates					
[Heme]_0_k_autox_ (mM/L/h)	0.081	0.170	0.477	0.024	0.835
[Heme]_0_ [NO]_0_k_NO_ (mM/L/h)^i^	149	176	343	54	478
[Heme]_0_ [NO]k_NO_ (mM/L/h)^j^ Steady State	∼0.01	∼0.01	∼0.01	∼0.01	∼0.01
[Heme]_0_k_half-life_	0.084	0.039	0.041	0.013	0.042

^a^ Weighted average doses from clinical trial data.

^b^ Initial average HBOC heme concentration in plasma obtained by multiplying the dose in g/kg by 1.47.

^c^ Calculated from half-life *versus* dose plots.

^d^ Calculated area under the HBOC concentration *versus* time function as described in Methods using the initial [Heme]_0._

^e^ From (Meng et al., 2018).

^f^ Calculated as 0.693/half-life.

^g^ From (Meng et al., 2018) converted to mM^-1^h^-1^.

^h^ Steady state NO concentration assuming a total endothelial NO secretion rate into plasma of 0.0236 mM/L/h.

^i^ Initial rate of reaction assuming a [NO]_0_ of 1 × 10−6 mM/L.

^j^ Assuming NO reaction rate with HBOC equals the NO secretion rate into plasma.

Rate constants for overall HBOC removal at the average administered dose (k_T_) were calculated as 0.693/T_1/2_, where T_1/2_ was calculated as described above. The results of these calculations are shown in Table 3.

### Comparison of MI ratio to pharmaceutical, biophysical, and biochemical parameters

The MI ratio for all five HBOCs or the CP HBOCs was graphically compared to various independent variables and evaluated for best fit to linear and quadratic functions utilizing the EXCEL data analysis package (Table 4).

**TABLE 4:** Comparison of MI ratios to various independent variables by regression analysis.^a^

Independent Variable	Fit Equation type	R^2^	Two tailed p value	y intercept
[Hb]_0_ (mM heme/L)^b^	Linear	0.9216	0.080	−2.06
	Quadratic	0.9994	0.049	2.86
R_g_ CP HBOCs (nm)^c^	Linear Quadratic	0.7354 0.9476	0.285 0.458	−2.47 7.68
R_g_ All HBOCs (nm)^c^	Linear Quadratic	0.8105 0.9649	0.074 0.070	−3.02 6.97
Half-life (h)^d^	Linear	0.8138	0.196	−0.84
	Quadratic	0.9961	0.125	2.15
AUC (mM/L)h^e^	Linear	0.9613	0.039	0.38
	Quadratic	0.9999	0.024	1.11
k_a_ (h^-1^)^f^	Linear	0.7304	0.291	−1.89
	Quadratic	0.9325	0.520	6.77
k_N_ (mM^-1^h^-1^)^g^	Linear	0.0586	0.484	−6.51
	Quadratic	0.2399	0.256	−308
k_T_ (h^-1^)^h^	Linear	0.3844	0.760	5.02
	Quadratic	0.8752	0.706	12.3
P_50_ (mmHg)^i^	Linear	0.1597	0.799	23.2
	Quadratic	0.9421	0.481	−7,656
[Hb]_0_k_a_ (mM/L/h)	Linear	0.9243	0.077	0.052
	Quadratic	0.9993	0.053	1.39
[Hb]_0_ [NO]k_N_ (mM/L/h)^j^	Linear	0.8836	0.120	−1.7459
	Quadratic	0.9973	0.103	4.1345
[Hb]_0_k_T_ (mM/L/h)	Linear	0.1711	0.827	5.8089
	Quadratic	0.8108	0.870	−132

^a^ All comparisons are between the MI ratio and the listed independent variable. All fits utilized the four CP HBOC data points with the exception of the All HBOCs R_g_ in which all five HBOC data points were utilized. Fits determined using an EXCEL data analysis package.

^b^ Average initial HBOC dose.

^c^ Average HBOC size as determined by published or estimated radius of gyration.

^d^ Half-life estimated for the average HBOC dose from the best linear fits to dose *versus* half-life data.

^e^ AUC values estimated as described in Methods assuming an exponential rate of HBOC removal from plasma and a quadratic dose dependence derived from the best linear fit to HBOC dose *versus* half-life plots.

^f^ Autoxidation rate constant from (Meng et al., 2018).

^g^ NO oxidation rate constant from (Meng et al., 2018).

^h^ HBOC removal rate constant calculated by dividing 0.693 with the estimated half-life.

^i^ Oxygen partial pressure at which HBOCs are half saturated from (Meng et al., 2018).

^j^ Reaction rate with NO assumes an initial NO concentration of 1 × 10−6 mM/L.

### Calculation of expected component AUC values

A mathematical model of the variation in time of the concentrations of reduced, autoxidized, and NO oxidized HBOCs was constructed assuming that autoxidation and overall HBOC removal could be described by first order rate constants and the reaction with NO was equal to the NO secretion rate. The contribution of methemoglobin reduction reactions was assumed to be negligible and the kinetics of overall HBOC removal from circulation was assumed to be the same for reduced and oxidized hemoglobin (Snyder et al., 1987; Vandegriff et al., 2006)). For simplicity, the starting metHBOC concentration was assumed to be zero. These assumptions yield the following rate equations:

$$d{\left[\text{Hb}\right]}_{r}/\text{dt}=‐{\left[\text{Hb}\right]}_{r}\left(k_{T}+k_{a}\right)\;–\;R$$

$$d{\left[\text{Hb}\right]}_{a}/\text{dt}=k_{a}{\left[\text{Hb}\right]}_{r}\;‐\;k_{T}{\left[\text{Hb}\right]}_{a}$$

$$d{\left[\text{Hb}\right]}_{N}/\text{dt}=R\;‐\;k_{T}{\left[\text{Hb}\right]}_{N}$$

where [Hb]_r_, [Hb]_a_, and [Hb]_N_ are the concentrations of reduced, autoxidized and NO oxidized HBOC, respectively; k_a_ and k_T_ are the first order rate constants for HBOC autoxidation and overall HBOC removal from plasma, respectively; and R is the rate of NO secretion into plasma.

The solutions to these equations are (Ritger and Rose, 1968):

$${\left[\text{Hb}\right]}_{r}=\left({\left[\text{Hb}\right]}_{0}+R/K\right)e^{‐\text{Kt}}\;–\;R/K$$ (2)

$${\left[\text{Hb}\right]}_{a}=\left({\left[\text{Hb}\right]}_{0}+R/K\right)\left(e^{{‐k}_{T}t}\;–\;e^{‐\text{Kt}}\right)+\left({\text{Rk}}_{a}/{\text{Kk}}_{T}\right)\left(e^{{‐k}_{T}t}\;–\;1\right)$$ (3)

$${\left[\text{Hb}\right]}_{N}=\left(R/k_{T}\right)\left(1\;–\;e^{{‐k}_{T}t}\right)$$ (4)

where K = k_a_ + k_T_. In integrating these equations to give the AUC for each HBOC, it is noted that Equation 2 will equal zero at a finite time, t_0_, given by:

$$t_{0}=\left(\ln\left(K{\left[\text{Hb}\right]}_{0}/R+1\right)\right)/K$$

Therefore, to calculate the AUC for [Hb]_r_, Equation 2 was integrated from t = 0 to t = t_0_ to yield:

$$\text{AUC }{\left[\text{Hb}\right]}_{r}=\left(\left({\left[\text{Hb}\right]}_{0}+R/K\right)\left(1\;–\;e^{{‐\text{Kt}}_{0}}\right)\;–\;{\text{Rt}}_{0}\right)/K$$

Time t_0_ is also the time at which the generation of autoxidized or NO oxidized HBOC will stop, since there is no more reduced hemoglobin substrate for these reactions. The concentrations of these species at t_0_, denoted as [Hb(t_0_)]_a_ and [Hb(t_0_)]_N_, respectively, are then assumed to decrease with a first order exponential decay with a k_T_ rate constant. The AUCs for [Hb]_a_ and [Hb]_N_ are therefore given by the integral of Equations (3) and (4) from t = 0 to t = t_0_ plus the integral of the exponential decay of these HBOC species from t = t_0_ to infinity:

$$\text{AUC}\;{\left[\text{Hb}\right]}_{a}=\left({\left[\text{Hb}\right]}_{0}+R/K\right)\,\left(\left(e^{{‐\text{Kt}}_{0}}\;–\;1\right)/K\;–\;\left(e^{{‐k}_{T}t_{0}}\;–\;1\right)/k_{T}\right)+\left({\text{Rk}}_{a}/{\text{Kk}}_{T}\right)\,\left(\left(1\;‐\;e^{{‐k}_{T}t_{0}}\right)/k_{T}\;–\;t_{0}\right)+{\left[\text{Hb}\left(t_{0}\right)\right]}_{a}/k_{T}$$

$$\text{AUC }{\left[\text{Hb}\right]}_{N}=R\left(e^{{‐k}_{T}t_{0}}/k_{T}+t_{0}\;–\;1/k_{T}\right)/k_{T}+{\left[\text{Hb}\left(t_{0}\right)\right]}_{N}/k_{T}$$

Note that for R = 0 these equations simplify to:

$$\text{AUC }{\left[\text{Hb}\right]}_{r}={\left[\text{Hb}\right]}_{0}/K$$

$$\text{AUC }{\left[\text{Hb}\right]}_{a}={\left[\text{Hb}\right]}_{0}\left(1/k_{T}\;–\;1/K\right)\text{ and}$$

$$\text{AUC }{\left[\text{Hb}\right]}_{N}=0$$

### Comparison of model predictions to AUCs determined from clinical data

To compare model predictions with actual clinical data, total HBOC and metHBOC AUC values were obtained using the data measuring tool in Adobe Acrobat applied to data from a dose escalation study of Hemospan (Olofsson et al., 2006). Model predicted values for these paraments were then calculated utilizing various autoxidation rate constants and NO secretion rates (Table 5). These calculations assumed an initial plasma heme concentration of 0.418 mM/L which was calculated to result from the infusion of the 0.27 g/kg HBOC, a dose used for two of the patient cohorts (n = 4 each) in this trial. These cohorts were chosen for comparison in part because the initial plasma HBOC concentration was closest to that of the 0.35 mM/L average estimated for all of the later stage Hemospan clinical trial data and because the use of two cohorts gives the largest number of total data points. Total AUC predictions were also compared with data published from a HemAssist clinical trial (O’Hare et al., 2001) in the same manner.

**TABLE 5:** Comparison of total and methemoglobin AUCs estimated from Hemospan clinical trial data and calculated using the mathematical model with different assumed values for NO secretion into plasma and autoxidation rate constants.

Data source	AUCs (mM/L)h^a^	
	Total Hb	metHb
Hemospan clinical trial	13.0 (8.5,17.5)	3.9 (3.0,4.7)
Mathematical model^b^		
R = 0.01, k_a_ = 0.07	10.9	8.6
R = 0.00, k_a_ = 0.07	10.9	7.1
R = 0.01, k_a_ = 0.00	10.9	6.7
R = 0.001, k_a_ = 0.007	10.9	3.2
R = 0.01, k_a_ = 0.021	10.9	7.5
R = 0.001, k_a_ = 0.021	10.9	4.9
R = 0.0001, k_a_ = 0.021	10.9	4.1
R = 0.0, k_a_ = 0.021	10.9	3.9

^a^ Data from (Olofsson et al., 2008) from two cohorts given an identical HBOC dose estimated to result in an initial plasma concentration of 0.418 mM/L (n = 4 patients per cohort); values are the average of those derived from integration of the appropriate concentration *versus* time data with the individual cohort values given in parentheses.

^b^ Calculated from pharmacokinetic model as described in Methods assuming a starting dose of 0.418 mM/L; R is the rate of NO secretion into plasma (mM/L/h); k_a_ is the autoxidation rate constant (h^−1^); the *in vitro* measured k_a_ values for Hemospan are 0.07 h^−1^ (Meng et al., 2018) and 0.021 (Vandegriff et al., 2006).

### Comparison of calculated AUCs with MI ratios

Estimated AUCs of reduced, autoxidized, NO oxidized, total, and total oxidized HBOC species were calculated for all five HBOCs at the average doses evaluated in clinical testing, using three combinations of autoxidation rate constants and NO secretion rates (Table 6). These AUC values were then compared to the corresponding HBOC MI ratios (Table 7).

**TABLE 6:** AUC values predicted by a mathematical model of HBOC oxidation and removal from plasma for differing autoxidation rate constants and NO secretion rates.

	HBOC				
AUC (mM/L)h^a^	HemAssist	Hemolink	Hemopure	Hemospan	Polyheme
R = 0.01, k_a_ = Meng^b^					
[Hb]_r_ ^c^	5.01	6.98	8.38	1.81	11.2
[Hb]_a_ ^d^	4.91	30.3	97.1	3.26	223
[Hb]_N_ ^e^	2.07	6.44	8.74	3.64	12.6
[Hb]_met_ ^f^	6.98	36.7	106	6.90	236
[Hb]_Total_ ^g^	12.0	43.7	114	8.72	247
R = 0.001, k_a_ = 0.1Meng					
[Hb]_r_	10.3	28.3	50.3	6.06	79.1
[Hb]_a_	1.00	12.3	58.2	1.09	158
[Hb]_N_	0.59	3.14	5.78	1.57	9.54
[Hb]_met_	1.58	15.4	64.0	2.66	167
[Hb]_Total_	11.9	43.7	114	8.72	247
R = 0.000, ka = 0.3Meng					
[Hb]_r_	9.23	19.0	25.5	5.67	35.3
[Hb]_a_	2.67	24.7	88.7	3.05	212
[Hb]_N_	0	0	0	0	0
[Hb]_met_	2.67	24.7	88.7	3.05	212
[Hb]_Total_	11.9	43.7	114	8.72	247

^a^ AUC values calculated for average clinical doses of each HBOC as denoted in Table 2; R is the NO secretion rate (mM/L/h), k_a_ is the autoxidation rate constant (h^−1^).

^b^ k_a_ values reported by (Meng et al., 2018).

^c^ AUC for reduced HBOC.

^d^ Autoxidized HBOC AUC.

^e^ AUC of HBOC oxidized by NO.

^f^ Sum of AUC [Hb]_a_ and AUC [Hb]_N._

^g^ Total AUC is the sum of AUCs for [Hb]_r_, [Hb]_a_, and [Hb]_N._

**TABLE 7:** Comparison of MI ratios to model predicted AUC values by linear regression analysis.^a^.

Independent Variable AUC (mM/L)h	Fit Equation type	R^2^	Two tailed p value	y intercept
Model for R = 0.01, k_a_ = Meng^b^				
[Hb]_r_ ^c^	Linear	0.8698	0.135	−4.77
	Quadratic	0.9994	0.051	7.24
[Hb]_a_ ^d^	Linear	0.9705	0.030	0.62
	Quadratic	0.9998	0.025	1.12
[Hb]_N_ ^e^	Linear	0.7794	0.234	−1.04
	Quadratic	0.9999	0.021	2.26
[Hb]_met_ ^f^	Linear	0.9664	0.034	0.52
	Quadratic	0.9998	0.030	1.11
[Hb]_Total_ ^g^	Linear	0.9649	0.036	0.38
	Quadratic	0.9997	0.032	1.09
Model for R = 0.001, k_a_ = 0.1Meng				
[Hb]_r_	Linear	0.8739	0.130	−0.57
	Quadratic	0.9992	0.055	1.67
[Hb]_a_	Linear	0.9878	0.012	0.89
	Quadratic	0.9999	0.015	1.15
[Hb]_N_	Linear	0.8673	0.137	−0.11
	Quadratic	0.9997	0.036	1.37
[Hb]_met_	Linear	0.9850	0.015	0.83
	Quadratic	0.9999	0.022	1.13
[Hb]_Total_	Linear	0.9649	0.036	0.38
	Quadratic	0.9997	0.032	1.09
Model for R = 0.000, k_a_ = 0.3Meng				
[Hb]_r_	Linear	0.8032	0.208	−1.94
	Quadratic	0.9993	0.052	3.47
[Hb]_a_	Linear	0.9744	0.026	0.71
	Quadratic	0.9999	0.023	1.14
[Hb]_N_	Linear	-	-	-
	Quadratic	-	-	-
[Hb]_met_	Linear	0.9744	0.026	0.71
	Quadratic	0.9999	0.023	1.14
[Hb]_Total_	Linear	0.9649	0.036	0.38
	Quadratic	0.9997	0.032	1.09

^a^ All comparisons are between the MI ratio and the listed independent variable. Fits determined using the EXCEL data analysis package.

^b^ R is the assumed NO secretion rate into plasma (mM/L/h); autoxidation rate constants (k_a_) are those reported by (Meng et al., 2018) or fractions thereof.

^c^ AUC of reduced HBOC.

^d^ AUC of autoxidized HBOC.

^e^ AUC of NO oxidized HBOC.

^f^ AUC of total oxidized HBOC calculated as the sum of AUCs of Hb_A_ and Hb_N_.

^g^ Total AUC calculated as the sum of the individual component AUCs.

### Statistical analysis

Incidence rate means and standard deviations for treated and control patients were calculated along with the 99% confidence intervals using a modified Wald method (Motulsky, 2010). Statistical data with respect to best fits of MI *versus* various parameters were taken from the EXCEL data analysis package. All p values are two-tailed obtained by doubling the values reported in the EXCEL analysis.

## Results

### Comparison of MI incidence ratio with HBOC dose, size, plasma half-life and AUC

**FIGURE 2:**
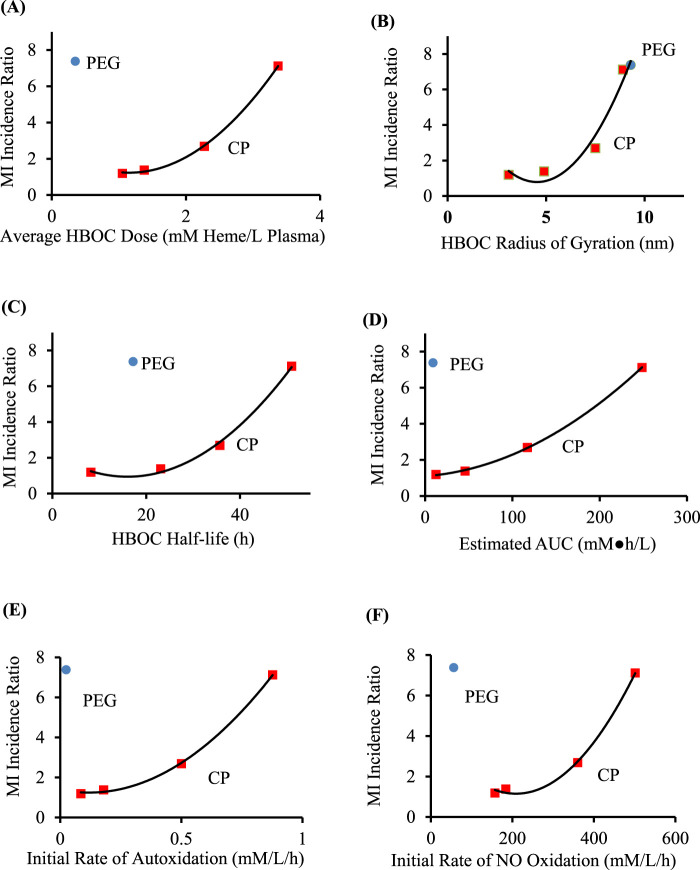
Comparison of MI ratios to average HBOC dose **(A)**, size **(B)**, circulatory half-life **(C)**, estimated AUC **(D)**, initial rate of autoxidation **(E)**, and initial rate of oxidation by NO **(F)**, for crosslinked and polymerized (CP) HBOCs (squares) and Hemospan, a PEG modified HBOC (PEG, circle). The equations of best fit (Table 4) are for the CP HBOCs (n = 4) except for the comparison with average molecular size (n = 5). Data are derived from clinical trials enrolling a total of 206–592 patients in each treated or control group for each HBOC.

HBOC properties (Table 1), pharmacokinetic data (Tables 2), and estimated *in vivo* reaction rates for oxidative reactions and overall HBOC removal from plasma (Table 3) were summarized, and comparisons made between MI incidence ratios and various independent variables by regression analysis (Table 4; Figure 2). There is a significant (R^2^ = 0.9994, p < 0.05) positive correlation between MI ratio and dose for the four CP HBOCs, with the best fit being a quadratic function. There is also a positive correlation (R^2^ = 0.9649) between MI and HBOC size. These two correlations confirm the results from a preliminary analysis performed with a smaller, less refined data set (Estep, 2019). Since HBOC size correlates positively with intravascular half-life over the molecular weight range encompassed by the evaluated HBOCs (Berbers et al., 1991; Bleeker et al., 1992; Keipert et al., 1992; Hsia et al., 1992; Conover et al., 1997; Wicks et al., 2003; Estep, 2015; Taguchi et al., 2017), MI incidence was compared with this parameter as well (Table 4; Figure 2C) using estimated HBOC half-lives derived from the best linear fits to published dose *versus* half-life data. Here again a positive correlation was observed, although this did not reach statistical significance (R^2^ = 0.9961, p = 0.12). Collectively, these data suggest that the total exposure of blood and endothelium to HBOCs as reflected in the area under the HBOC plasma concentration *versus* time curve (AUC) is important, since dose and intravascular persistence are the primary determinants of AUC. No AUC data were reported for HBOCs in Phase II or III clinical trials, but this parameter can be estimated as described in Methods as a quadratic function of dose (Table 3). When MI incidence is compared with these AUC values (Table 4; Figure 2D), the correlation for the CP HBOCs (R^2^ = 0.9999, p = < 0.03) is stronger than with either dose, size or half-life alone. Note also that, by virtue of how the MI ratio is defined, the y intercept should approach 1.0 as dose or AUC approach zero. The y-intercept (1.11) with the AUC *versus* MI ratio function agrees closely with this expectation. In these comparisons PEG HBOC exhibits a notably higher incidence of MI than CP HBOCs at comparable values of dose or AUC.

### Comparison of MI incidence ratio with HBOC biochemical and biophysical properties

To explore potential toxicity mechanisms, MI ratios were compared to several HBOC properties, as well as calculated initial average reaction rates in plasma (Table 4). No significant correlations were observed between the MI ratio and HBOC oxygen half saturation values (P_50_), or the rate constants for autoxidation, reaction with nitric oxide (NO), or overall HBOC removal from circulation, or the initial rate of overall HBOC removal from circulation. Positive correlations between MI and initial autoxidation and NO oxidation rates were high, although they did not reach statistical significance (Table 4; Figures 2E,F). It is also recognized that the initial oxidation rate by NO would only persist for a short period of time because the millimolar concentration of HBOC would rapidly consume NO, driving this concentration down to sub picomolar levels ([NO]_ss_, Table 3). At this point the reaction rate of HBOC with NO would be expected to equal the rate of NO secretion into plasma. However, due to the fact that the NO reaction rate constant is approximately nine orders of magnitude greater than the autoxidation or hemoglobin removal rate constants, the reaction rate of HBOC oxidation with NO is still comparable to the rates of autoxidation and HBOC plasma clearance, especially at lower HBOC concentrations (Table 3).

### Calculation of AUCs for various HBOC species

By analogy with the concept of AUC for the total HBOC concentration, one can also contemplate assessing the relationship between the MI ratio and the AUCs of the products of the two primary HBOC oxidation reactions, autoxidation and oxidation by reaction with NO. Both reactions generate oxidized hemoglobin (metHBOC), with the former reaction also generating superoxide and the latter resulting in the consumption of NO. While it is recognized that metHBOC formed by these two processes cannot be distinguished experimentally, one can conceptually model the contributions to HBOC oxidation by these two reactions with the sum resulting in the experimentally accessible total metHBOC concentration. Therefore, a mathematical model was constructed to predict the expected evolution of these reaction products with time. Key assumptions were that the primary mechanisms for reduced HBOC disappearance are autoxidation, NO oxidation, and overall HBOC removal, with the last of these acting equally on both reduced and oxidized HBOCs (Snyder et al., 1987; Vandegriff et al., 2006). It was further assumed that no oxidized HBOC (metHBOC) is converted to the reduced form, that doses of HBOC were administered as a bolus, and that the reaction rate of HBOC with NO is equal to the rate of NO secretion into plasma. Equations for the concentration of these HBOCs were integrated to provide estimates of their respective AUCs. Several different assumed values for the autoxidation rate constants and NO secretion rates were explored (Tables 5, 6).

### Comparison of model predictions with clinical data

To assess the veracity of the mathematical model, predicted AUC values were compared to those calculated from published pharmacokinetic data for Hemospan (Olofsson et al., 2008), as this is the only data set of which the author is aware in which plasma concentrations of both total and metHBOC were reported in detail. Model calculations assumed an initial plasma heme concentration of 0.418 mM/L, corresponding to the dose administered to two cohorts in this dose-response study (Table 5). The integrated clinical data yield AUCs of 8.48 and 17.5 mM•h/L for the two cohorts, a difference which probably reflects the inherent biological variability in these small (n = 4) sample sizes. The predicted total AUC of 10.9 mM•h/L from the mathematical model is between the two values measured from these cohorts, and similar to their average (13.0 mM•h/L), suggesting reasonable agreement. However, the degree of total oxidation predicted by the mathematical model (8.6 mM•h/L) utilizing the data of Meng et al. (Meng et al., 2018) is significantly greater than that observed in the clinical data (3.0 and 4.7 mM•h/L). One possibility is that the difference is a result of lower *in vivo* oxidation rates compared with those measured *in vitro*. To explore this possibility, expected values for total oxidized HBOC were calculated assuming that either the autoxidation rate constant or the NO secretion rate were zero. Although the total predicted oxidized HBOC AUC was reduced, in neither case was it reduced to the value measured *in vivo* (Table 5). Only when both the autoxidation rate constant and the NO secretion rate were simultaneously reduced by a factor of ten did the predicted total metHBOC AUC (3.2 mM•h/L) agree within experimental error with clinical observations. However, other combinations of reductions in autoxidation rates and NO secretion rates are also possible, and the developers of Hemospan reported an autoxidation rate constant approximately 0.3 that of Meng and coworkers (Vandegriff et al., 2006). When this rate constant was combined with the assumption of negligible NO secretion into plasma, good agreement was obtained with the observed total methemoglobin AUC for Hemospan (3.9 mM•h/L for both calculated and measured average values).

The only other clinical data set containing a sufficient number of observations to perform an AUC integration with confidence was a report of the total plasma hemoglobin concentration of HemAssist (O’Hare et al., 2001). The total AUC value predicted from the model (22 mM•h/L) is similar to, but somewhat higher than, that estimated from the clinical data (16 mM•h/L). The absence of sufficient plasma metHBOC values from this and other reported clinical data preclude further comparisons.

### Comparison of MI values with predicted AUCs

In light of the results of comparison of the model predictions with Hemospan clinical data, estimated AUC values for the different HBOC components were calculated using three different assumptions for the autoxidation rate constants and NO secretion rates (Tables 6 and 7). The total AUC values predicted by this model are virtually identical to those predicted by the simpler method based on a quadratic function of dose as described in Methods (compare AUC values in Tables 3, 6). The total AUC also does not change when the assumed NO secretion rate and/or the autoxidation rate constants are multiplied by the same factor (Table 6), but the AUC values for differing HBOC components (i.e., reduced, autoxidized, and NO oxidized) do.

**FIGURE 3:**
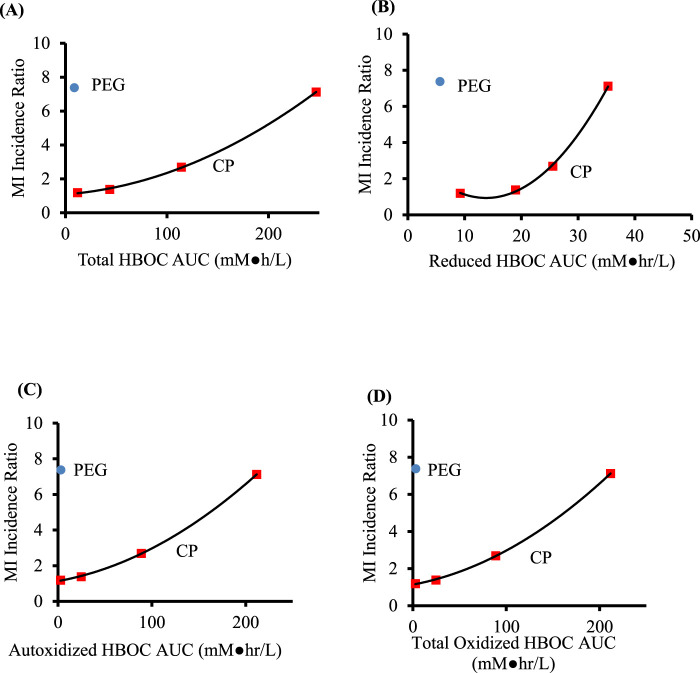
Comparison of AUC values predicted from a mathematical model of HBOC pharmacokinetics for crosslinked and polymerized (CP) HBOCs (squares) and Hemospan, a PEG modified HBOC (PEG, circle). Calculations assumed autoxidation rate constants 0.3 that reported by (Meng et al., 2018) and a zero NO secretion rate. Specific AUCs are for total **(A)**, reduced **(B)**, autoxidized **(C)**, and total oxidized **(D)** HBOCs. The adjusted coefficients of determination, p values, and y intercepts for equations of best fit for the CP HBOCs are given in Table 7. Data are derived from clinical trials enrolling a total of 206–592 patients in each treated or control group for each HBOC.

Good correlations between the MI ratios and estimated AUCs are obtained for reduced, autoxidized, NO oxidized, total oxidized and total HBOC for both scenarios in which a finite NO secretion rate is assumed, and for each of the AUCs save that for total NO oxidized hemoglobin in the scenario in which NO secretion is assumed to be zero (Table 7, Figure 3). However, taking into account y-intercept values as well as the correlation coefficients and p-values, best agreement is observed with quadratic fits between the MI ratios and autoxidized, total methemoglobin, and total HBOC AUCs.

## Discussion

A narrative has been promoted in the HBOC literature that toxicity is primarily a consequence of the extravasation of lower molecular weight hemoglobins and their consumption of NO in the interstitial space, thereby causing vasoconstriction (Gould et al., 1998). While there is significant experimental support for this process as a primary mechanism for HBOC induced hypertension (Olson et al., 2004), this narrative ignores the potential adverse consequences of intravascular interactions. It also implies that HBOCs of larger size should be less toxic; however, the opposite is observed with respect to MI. Furthermore, infusion of HBOCs into rats, dogs, cats, or pigs resulted in no decrease in coronary blood flow (Sharma et al., 1994; Kingma et al., 2002; Ulatowski et al., 1996; Mongan et al., 2009), even in the face of substantial overall increases in systemic vascular resistance (Sharma et al., 1994), nor were coronary artery dimensions decreased in human cardiac patients after HBOC infusion (Collins et al., 1993; Serruys et al., 2008; Meliga et al., 2008). This implies that other mechanisms are important in increasing MI risk and that these are intravascular in nature.

The fact MI incidence exhibits a quadratic dependence with respect to HBOC dose motivated exploration of why this should be the case. This ultimately led to the realization this is at least in part a consequence of the fact that HBOC AUCs also exhibit a quadratic dose dependence, since increasing dose increases the circulatory half-life as well as the initial value of plasma HBOC concentration. Indeed, the correlation of MI ratio with AUC is stronger than that with either dose or size, suggesting that AUC is the better basis for the interpretation of biological effects.

HBOCs exacerbate oxidative stress in a variety of cellular, tissue and whole animal models with an important first step being an autoxidative process in which bound oxygen dissociates as superoxide, leaving the hemoglobin in the oxidized (methemoglobin) form (Alayash, 2019). Both superoxide and methemoglobin may participate in oxidative stress reactions, some of which are known to cause endothelial dysfunction (D’Agnillo, 2013) and increased vaso-occlusion, with accelerated heme loss from methemoglobin identified as a major contributing factor (Belcher et al., 2014). The oxidation of HBOCs in plasma to methemoglobin has been directly observed in clinical trials (Sprung et al., 2002; Olofsson et al., 2006; Olofsson et al., 2008; O’Hare et al., 2001). While humans may have some capacity to reduce plasma methemoglobin (Vandegriff et al., 2006; McGown et al., 1990), this capacity is limited and may be overwhelmed in patients with pre-existing endothelial dysfunction at higher HBOC doses (Biro, 2012; D’Agnillo, 2013). Oxidative stress is also variable among patients, with an extensive literature correlating such stress with increased MI risk (Wang and Kang, 2020; Liguori et al., 2018). In light of this, it is not surprising the incidence of MI is highly correlated with the initial rate of CP HBOC autoxidation as well as the estimated autoxidation AUC. Indeed, the correlation between HBOC infusion, oxidative events and enhanced MI incidence may be the most rigorous proof to date of the hypothesis that oxidative stress increases MI risk.

Methemoglobin is also formed when hemoglobin reacts with NO, with conversion of the latter to nitrate (D’Agnillo, 2013). Comparing the amount of metHBOC generated by autoxidation *versus* NO oxidation as reflected in their estimated relative AUC values suggests the former is more important at higher HBOC doses, but the latter can make a significant contribution at lower doses (Table 6). What cannot be directly compared in this analysis is the relative contribution of oxidative stress resulting from metHBOC formation and the consequences of profound NO depletion. For example, NO consumption is known to activate platelets (Radomski et al., 1987) and could contribute to endothelial dysfunction (Biro, 2012; D’Agnillo, 2013). Ideally, both the autoxidation and NO reaction rate constants should be reduced to minimize HBOC toxicity.

In comparing MI risk enhancement between CP HBOCs and Hemospan at comparable doses, the latter increases risk to a greater extent. This may be a consequence of increased tetramer dissociation in PEG HBOCs (Caccia et al., 2009) which can in turn accelerate autoxidation (Zhang et al., 1991). However, the *in vitro* measured autoxidation rate constant for Hemospan is actually slightly lower than that of the CP HBOCs (Meng et al., 2018). A more likely possibility is that PEG modification of hemoglobin leads to an increased rate of heme loss, which can in turn exacerbate vaso-occlusion (Belcher et al., 2014). Meng et al. reported that the fast phase of heme loss from Hemospan was greater than that of CP HBOCs and approximately 1.4 -fold greater than that of unmodified human hemoglobin (Meng et al., 2018). Vandegriff and coworkers reported that the fast phase of heme loss from Hemospan was fivefold greater than that of unmodified hemoglobin (Vandegriff et al., 2006). Free heme has been increasingly implicated as contributing to endothelial dysfunction and MI risk (Guo et al., 2022). Thus, although PEG modification has proven useful in improving the therapeutic index of other proteins, it may not be helpful in improving the desired characteristics of HBOCs.

Some may be disconcerted by the fact that multiple combinations of autoxidation rate constants and NO secretion rates result in AUC values that demonstrate a strong correlation to the MI ratios (Tables 5–7). However, this reflects the mathematical reality that multiplication of any set of independent variables by the same constant will not affect the correlation coefficient due to the fact that the independent variables appear in both the numerator and denominator to the same power. Thus, correlations do not in themselves necessarily reflect the relative absolute contributions of different variables to the observed dependent variable. This can be better defined by comparison of the absolute values of AUC to actual clinical data as was done with respect to Hemospan. Unfortunately, this is the only HBOC clinical data set which has a sufficient number of plasma metHBOC data points in order to do so. For this reason, correlations of various AUC values with MI ratios were assessed for a range of autoxidation and NO secretion rates, encompassing the specific values that give good agreement with clinical data for Hemospan. The fact that there were strong correlations between AUC values and MI incidence in all of these scenarios implies that these correlations are robust in this sense.

The analysis presented in this publication is limited by several factors. None of the clinical trials from which data were derived were specifically designed to explore possible mechanisms by which HBOCs may increase MI risk, in part because this was not identified in preclinical studies or individual clinical trials as a product related adverse event prior to 2008. It was only when data are aggregated that this became apparent (Natanson et al., 2008). In addition, the diagnosis of MI is not necessarily straightforward in the presence of HBOCs because hemoglobin can interfere with MI diagnostic troponin assays (Estep, 2019), although several HBOC developers have considered such interference (Ma et al., 1997; Olofsson et al., 2011). The exact criteria used for MI diagnosis are also not always well described, and may therefore differ, which could contribute to between study variability. On the other hand, it is reasonable to assume that the same criteria are used in assessing treated and control patients within a study, and common authorship across a number of the studies should increase consistency. Finally, the analyses presented in this publication required the estimation, interpretation or extrapolation of a variety of parameters. The need for data aggregation also results in only four or five data points for each comparative analysis. As a consequence, the observations presented in this publication should be considered as hypothesis generating rather than definitive.

Collectively, the analyses presented in this communication, when viewed in light of an extensive literature on HBOC safety, suggests that efforts to minimize hemoglobin oxidative reactions should result in less toxic formulations (Bian and Chang, 2015). In addition, further exploration of the manner in which HBOCs impact patient oxidation/reduction status and endothelial dysfunction could usefully improve patient selection criteria. Such efforts may ultimately enable the identification of patient subpopulations at low risk for HBOC side effects.

## Data Availability

The raw data supporting the conclusions of this article will be made available by the authors, without undue reservation.
